# How home HIV testing and counselling with follow-up support achieves high testing coverage and linkage to treatment and prevention: a qualitative analysis from Uganda

**DOI:** 10.7448/IAS.19.1.20929

**Published:** 2016-06-28

**Authors:** Norma C Ware, Monique A Wyatt, Stephen Asiimwe, Bosco Turyamureeba, Elioda Tumwesigye, Heidi van Rooyen, Ruanne V Barnabas, Connie L Celum

**Affiliations:** 1 Department of Global Health and Social Medicine, Harvard Medical School, Boston, MA, USA; 2 Division of Global Health Equity, Department of Medicine, Brigham & Women's Hospital, Boston, MA, USA; 3 Harvard Global, Cambridge, MA, USA; 4 Kabwohe Clinical Research Centre, Kabwohe, Uganda; 5 Human Sciences Research Council, Pretoria, South Africa; 6 Department of Global Health, School of Medicine, School of Public Health, University of Washington, Seattle, WA, USA

**Keywords:** HIV prevention, HIV treatment, linkage, home HIV testing and counselling, follow-up support, community-based HIV services, lay counsellors, Uganda

## Abstract

**Introduction:**

The successes of HIV treatment scale-up and the availability of new prevention tools have raised hopes that the epidemic can finally be controlled and ended. Reduction in HIV incidence and control of the epidemic requires high testing rates at population levels, followed by linkage to treatment or prevention. As effective linkage strategies are identified, it becomes important to understand *how* these strategies work. We use qualitative data from The Linkages Study, a recent community intervention trial of community-based testing with linkage interventions in sub-Saharan Africa, to show how lay counsellor home HIV testing and counselling (home HTC) with follow-up support leads to linkage to clinic-based HIV treatment and medical male circumcision services.

**Methods:**

We conducted 99 semi-structured individual interviews with study participants and three focus groups with 16 lay counsellors in Kabwohe, Sheema District, Uganda. The participant sample included both HIV+ men and women (*N*=47) and HIV-uncircumcised men (*N*=52). Interview and focus group audio-recordings were translated and transcribed. Each transcript was summarized. The summaries were analyzed inductively to identify emergent themes. Thematic concepts were grouped to develop general constructs and framing propositional statements.

**Results:**

Trial participants expressed interest in linking to clinic-based services at testing, but faced obstacles that eroded their initial enthusiasm. Follow-up support by lay counsellors intervened to restore interest and inspire action. Together, home HTC and follow-up support improved morale, created a desire to reciprocate, and provided reassurance that services were trustworthy. In different ways, these functions built links to the health service system. They worked to strengthen individuals’ general sense of capability, while making the idea of accessing services more manageable and familiar, thus reducing linkage barriers.

**Conclusions:**

Home HTC with follow-up support leads to linkage by building “social bridges,” interpersonal connections established and developed through repeated face-to-face contact between counsellors and prospective users of HIV treatment and male circumcision services. Social bridges link communities to the service system, inspiring individuals to overcome obstacles and access care.

## Introduction

The successes of HIV treatment scale-up and the availability of new prevention tools have raised hopes that the epidemic can be controlled and ended [1,2]. Reduction in HIV incidence requires high testing rates at population levels, followed by linkage to HIV treatment and effective prevention interventions. In sub-Saharan Africa (SSA), community mobilization and community-based testing have greatly expanded testing coverage [3–11]. However, the number of individuals making the link to services has been consistently suboptimal [7,12–18].

High linkage rates are achievable in Africa, as evidenced by a recent multi-site, randomized community intervention trial carried out in South Africa and Uganda. The Linkages Study evaluated community-based testing, coupled with a variety of linkage interventions. Results revealed high rates of linkage to clinic-based services for HIV-infected persons who received lay-counsellor follow-up support (more than 90%). For uninfected men, the uptake of referrals for medical circumcision was 47% among men who received follow-up support. Thirty-seven percent of infected persons initiated antiretroviral therapy (ART) in the two countries; of these, more than 80% were virally suppressed at nine months [19].

As effective linkage strategies are identified, it becomes important to understand *how* these strategies work. Answers to “how questions” are necessary to inform implementation and replication of positive trial results [20]. This paper addresses a “how question” in the context of The Linkages Study. Using qualitative data collected in conjunction with the trial, we ask how home HIV testing and counselling (home HTC) carried out by lay counsellors, combined with counsellor follow-up support, leads to high rates of linkage to clinic-based HIV treatment and prevention services. The larger goal is to shed light on the “mechanism of effect” of community-based HIV testing and linkage interventions [21].

## Methods

### Overview of The Linkages Study

The Linkages Study was a randomized trial to evaluate community-based HIV testing and linkage interventions [19]. Individuals testing positive and not already prescribed ART were randomized to one of two follow-up support interventions, or a control condition. Uncircumcised men testing negative were randomized to one of two follow-up support interventions intended to stimulate demand for medical male circumcision (MMC), or a control condition. “Lay counsellors,” members of local communities trained in HIV testing, counselling, follow-up and community mobilization techniques, implemented the testing and linkage interventions. Almost all of the lay counsellors were men (Table 2).

Counselling at HIV testing included: (1) information on the testing procedure, (2) HIV education and (3) explanation of the test result. Individuals testing HIV-positive were randomized 1:1:1 to (1) lay counsellor home visits, (2) clinic visit facilitation (help in navigating the initial clinic visit) or (3) the control condition of referral to a local HIV clinic. All HIV-negative uncircumcised men received information about the meaning and benefits of MMC at testing, and were randomized follow-up support strategies of (1) home visits, (2) text message reminders or (3) the control condition of promotion of MMC at the testing visit. Each participant in the home visit arms was visited by the same counsellor who had carried out his or her HIV testing. Home visits for follow-up support elicited responses to the experience for those who had made the link to HIV care or MMC services, and addressed challenges for those who had not. The text message reminder for MMC was intentionally short, to ensure compatibility with inexpensive mobile phones, and generic, to preserve confidentiality. The text read “Act Now!” in the local language (Runyankore).

The Linkages Study was carried out in rural KwaZulu-Natal, South Africa, and in Kabwohe, Sheema District, rural southwest Uganda. The qualitative research reported here was carried out at the Uganda site. Results from South Africa are reported elsewhere [8,22,23].

### Sampling and recruitment

Purposeful sampling is a qualitative sampling strategy aimed at in-depth understanding of a topic of investigation, rather than generalizing to larger populations [24]. To facilitate in-depth understanding, purposeful sampling entails selecting cases that are not only information-rich, but that reflect variation with respect to the topic under study. In this qualitative study, the topic is *home HTC combined with follow-up support by lay counsellors*. The criterion of information richness is met through the sampling of individuals with direct, topic-relevant experience: (1) individuals who received The Linkages Study intervention and (2) counsellors who implemented the intervention. The criterion of variation is met through sampling trial participants at different points in the intervention trial follow-up period (one to three months, 8 to 12 months, more than 12 months). To maximize information richness and variation, we sampled and included in the study as many participants as possible during the trial follow-up period. All lay counsellors were invited to participate.

### Qualitative data collection

Qualitative data collection consisted of individual interviews with the trial participant group (*N*=99), and three focus groups with 16 lay counsellors.

Individual interviews were semi-structured, using open-ended questions to systematically cover the following topics: (1) the home HTC experience; (2) responses to test results; (3) subsequent plans and/or efforts to access HIV care or prevention services; (4) outcomes of these plans or efforts; (6) experiences of follow-up support and (7) HIV education and support available in local communities. Ugandan research assistants (RAs) trained in qualitative research methods conducted the interviews in private settings, in the local language (Runyankore). Interviews lasted 45–60 minutes, and were audio-recorded.

Focus groups elicited the perceptions of barriers and facilitators to linkage to care and prevention services in communities, and the experiences of carrying out community mobilization and follow-up support. Each lasted about an hour. Focus groups were led by the primary and second authors (NCW, MAW). They were conducted in English and Runyankore, with translation provided by a Ugandan RA.

### Data quality

Audio-recordings of interviews and focus groups were transcribed in English by the RAs. The audio-recordings were translated by the same RAs who conducted the interviews, as part of the transcription process. Transcripts were reviewed for quality and used to provide continuous feedback on interview content and technique, and on detail and formatting of the transcription.

### Analysis

An inductive, content analytic approach was used to develop concepts and propositions from the qualitative interview and focus group data, beginning with data reduction [25]. To reduce the data, each interview transcript was summarized, creating a “testing-and-linkage-story” for each participant. Summaries and focus group transcripts were reviewed to identify emerging themes (repeated content) suggesting the “key ingredients” of lay counsellor home HTC with follow-up support. Themes were then grouped inductively to develop more general concepts and a set of framing propositional statements through which to represent core study results. These statements highlight and elaborate the functions of the key ingredients of home HTC and follow-up support – counselling interactions, home visits and lay counsellors from local communities. The propositions are illustrated using excerpts from qualitative data. Together, the propositions and elaborations show how the community-based HIV testing and linkage intervention led to linkage to clinic-based HIV treatment and prevention services.

### Ethical statement

Approval to carry out the qualitative research was obtained from the Human Subjects Division of the University of Washington, and the Uganda National Council for Science and Technology. Participants provided consent for the qualitative interviews as part of the consent process for The Linkages Study. Consent was re-confirmed verbally as part of recruitment for the qualitative study.

## Results

### Qualitative sample characteristics

Of 368 Ugandan trial participants receiving home HTC, 199 were sampled, 128 were located and invited to participate and 99 agreed. The participant sample included two subgroups: (1) individuals testing HIV-positive at home HTC and assigned to follow-up support or control (*N*=47) and (2) uncircumcised men testing HIV-negative in home HTC and assigned to follow-up support for linkage to MMC, or to control (*N*=52). Women made up almost two-thirds (64%) of the HIV-positive subgroup. The median age of the HIV-positive group was 33 years; median age of uncircumcised men was 26.5 years. In both subgroups, the majority of participants were married. Additional information appears in Table 1.

**Table 1:** Personal and study-related information on trial participants in the qualitative research (*N*=99)

	Total sample	HIV-positive persons: linkage to HIV treatment and care (*N*=47)	HIV-negative, uncircumcised men: linkage to MMC (*N*=52)
Personal information			
Gender (*N*=99)			
Women	30 (30%)	30 (64%)	
Men	69 (70%)	17 (36%)	52 (100%)
Age at trial enrolment (*N*=98) (median, IQR)	30 (24–37.75)	33 (26–40)	26.5 (22.75–32.25)
Relationship status (*N*=92)			
Never married	26 (28%)	3 (7%)	23 (44%)
Currently married	50 (55%)	22 (55%)	28 (54%)
Separated/divorced	12 (13%)	11 (28%)	1 (2%)
Widowed	4 (4%)	4 (10%)	0 (0%)
**Study-related information**			
Trial intervention arm (*N*=99)			
counsellor follow-up visits	37 (38%)	16 (34%)	21 (40%)
Clinic accompaniment	21 (21%)	21 (45%)	0 (0%)
SMS text-messages	28 (28%)	0 (0%)	28 (54%)
Control	13 (13%)	10 (21%)	3 (6%)
Months since trial Enrolment at qualitative interview (*N*=98)			
1–3 months	43 (44%)	32 (70%)	11 (21%)
8–12 months	24 (24%)	9 (19%)	15 (29%)
> 12 months	31 (32%)	5 (11%)	26 (50%)
Linkage at trial exit (*N*=98)		In care: 46 (100%) On ART: 26 (57%)	Circumcised: 22 (42%)

Fourteen of the 16 participating lay counsellors were men (88%); their median age was 33 years. Twelve (75%) were married. This information appears in Table 2.

**Table 2:** Personal information on qualitative research lay counsellor participants (*N*=16)

Personal information	
Gender (*N*=16)	
Women	2 (12%)
Men	14 (88%)
Median age at beginning of trial (*N*=15)	33 (32–34)
Relationship status (*N*=16)	
Married	12 (75%)
Single	4 (25%)

### Qualitative results

#### Overview

Trial participants endorsed HIV treatment and prevention in general. Immediately following home HTC, they expressed interest in seeking available services. HIV-infected individuals wanted treatment so as to live longer, healthier lives and continue to provide for their children. Relief and gratitude at receiving a negative test result prompted uninfected men to commit to remaining free of HIV.

Initial interest in linkage was eroded, however, by the difficulties of actually visiting a clinical care site. The expense of transport, time lost from income-generating activities, lingering fears and misunderstandings about HIV and MMC, uncertainties about how to manage a formal clinic visit, and the continuing, competing demands of daily life combined to leave people feeling demoralized, disconnected from the health care system and doubtful about the value and importance of investing in the effort to access care.

Follow-up support intervened to renew interest in linkage and inspire action. Different components contributed in different ways. Counselling interactions at testing and follow-up improved morale while also providing information about HIV. Repeated home visits were understood as signs of caring, creating a desire to reciprocate by seeking services. Lay counsellors from local communities as intervention implementers helped overcome fears and provide reassurance of services’ trustworthiness. These functions of home HTC and follow-up support are presented in greater detail in A–C below.

### A. Counselling at testing and follow-up improves morale while also communicating information about HIV

Communication of accurate information about HIV infection, treatment and prevention forms the core of counselling interactions in both testing and follow-up support. However, other dimensions are also important, and enhance the quality of the experience when they are managed well. Three additional dimensions emerged from interviewees’ accounts of their Linkages Study counselling experiences, as follows.

#### Respect

Interviewees cited a number of ways in which they felt treated with respect by lay counsellors. The counsellors were unfailingly polite. They were frank in communicating HIV test results, even when results indicated the presence of HIV infection. They were careful to honour the confidentiality of the testing and counselling encounter. And they took their time, allowing all of recipients’ questions to be answered:

The counsellor gives you all the time you want. At the clinic, you find many people and the counsellor wants to attend to all of them. But for this one, we met at our homes and he gives you all the time you need. – Man, aged 44

#### Encouragement

Feeling encouraged was critical to the counselling experience, as recounted by interviewees. Multiple types of communications from counsellors fall into this category. “Re-framing” refers to re-defining HIV as a chronic illness rather than a fatal disease, thereby reducing the threat. “Normalizing” is similar, reducing the threat of HIV by comparing it to a minor ailment, for example, a headache. Direct reassurance is a third type of encouragement highlighted by interviewees.

He [counsellor] treated me very well. We entered my house and he explained to me and counselled me and told me not to get worried. My heart got settled. And I said, “tomorrow I will go out and get treatment”. – Man, aged 38

#### Orientation and guidance

Orientation and guidance refers to the provision of specific instruction on how to access services as part of the counselling interaction. Interviewees stressed the value of being told where services were available, where the sites were located, how to get there, and when to go. Setting a date for a clinic visit as part of home HTC is part of orientation and guidance, as is being greeted upon arrival at clinic, being introduced to staff, and being accompanied by the counsellor to the visit starting point. Interviewees described the significance of orientation and guidance this way:

[*Orientation*] really helped me because [*the counsellor*] showed me someone to open a file for me and start getting medicine there. If I had come alone, I would not have managed, because I had never come there before. – Man, aged 38

Counselling interactions that communicate respect, encouragement and specific guidance on service seeking along with information, have an energizing effect. Recipients emerge from these encounters feeling strengthened and hopeful about the future. Improved morale means people feel more capable of making the link to services. One interviewee observed:

When I saw the way they were talking to me, it put me in a positive frame of mind. They showed me a right way to take care of my life. They really counselled me and I felt very hopeful. That is why I was able to reach [the clinic] here. – Woman, aged 38

This function was corroborated by lay counsellors, one of whom remarked that as a result of counselling:

All the people know these services. They know what they mean and they love them a lot. – Lay counsellor focus group participant, aged 33

### B. Repeated home follow-up visits are understood as signs of caring, creating a desire to reciprocate

Repeated home follow-up visits to promote linkage were a core activity of follow-up support in The Linkages Study. Visits served as “reminders” to link, but they also fulfilled broader functions.

Recipients of home visits understood them as signs of “caring” by counsellors and the organization they were understood to represent. This understanding is consistent with the meaning of “visiting” in social life in African communities. A visit requires effort. Visitors extend themselves for someone else, investing time and resources for another's benefit that could have been used to meet their own needs. In this way, visits signify caring, as reflected in the local saying, “you show you care with your feet.” The interviewee quoted below was drawing on the larger social meaning of visiting when she said:

Whenever [counsellor] visited me and counselled me, I felt loved. – Woman, aged 34

Because they signal caring, visits personalize the experience of services. Repeated visits create a sense of personal obligation to reciprocate, as personal acts of care must be repaid in kind. A way to repay home visits in which individuals were requested to seek health services was to act to fulfil the request. This dynamic is clearly expressed in the following statement, made by a man being visited to promote linkage to MMC:

I want to circumcise to make the counsellor happy. He keeps coming. He came like three times. I said, “ok, when I see him [*next*] I will ask him when they are doing circumcision. And I [*will*] go there and circumcise.” – Man, aged 39

### C. Lay counsellors from local communities help overcome fears and provide reassurance of services’ trustworthiness

The past decade has seen reduced fear of HIV in this rural Ugandan region, as the population has come to understand effective treatment is available. Fears and suspicions re-assert themselves, however, when people feel disconnected from services. “Fear talk” is heard, and worries about “tablets” (ART), disclosure of HIV-status, and resulting stigma are re-visited.

Because it is relatively new and unfamiliar, fears surrounding MMC can be acute. Men feared the procedure would be painful, and that unmanageable complications might ensue. They feared disruption of their sex lives and resulting marital disharmony. As most men in the region are subsistence farmers and/or work for a daily wage, absence of income during recovery from MMC was also a major concern. Male interviewees being visited to promote MMC readily shared their fears of circumcision:

The way my friends used to talk about it, we all feared to circumcise. We used to say “a person cannot be cut like a cow.” We saw circumcising as hard thing to be done. – Man, aged 23

As members of local communities, lay counsellors were well placed to help overcome fears. They were highly regarded; some held leadership positions in their towns and villages. They had proven themselves capable of providing valuable services to their communities. Because of who they were and what they did, they were trusted. One counsellor put it this way:

The people in the villages know us. They trust us because we are educated. They trust us because of how we conduct ourselves. They see what we are doing for them. – Lay counsellor focus group participant, aged 33

Services represented by trusted members of the community will themselves be seen as trustworthy. Confidence in the trustworthiness of services reassures people that seeking services will benefit themselves and their families. Greater perceived benefits help justify an investment of scarce resources in seeking care.

These three functions of lay counsellor home HTC with follow-up support built links to clinic-based services. Counselling that improved morale while also communicating information left participants feeling more confident, able to face up to the need for services and act effectively to overcome obstacles. Visits that generated a desire to reciprocate for the efforts and investment of counsellors made linking a way of acting in concert with local understandings and standards of behaviour. Lay counsellors who represented services while also being members of local communities *embodied* ties to the service system. These functions worked to increase individuals’ general sense of their personal capability, while also making the idea of services more manageable and familiar, thus reducing linkage barriers.

## Discussion

High rates of HIV testing and subsequent linkage to treatment and prevention services can be achieved in Africa. Community-based testing with follow-up by lay counsellors resulted in linkage for >90% of HIV-infected persons and ~50% of uninfected men in The Linkages Study. The demonstrated effectiveness of this combination makes it an appropriate context in which to take the next step of unpacking the intervention to show *how* it produces its effect. To this end, this qualitative analysis has highlighted and drawn out the functions of each of the three key ingredients of lay counsellor home HTC with follow-up support – counselling interactions, home visits and lay counsellors from local communities. We have characterized these functions as (1) improvement of morale; (2) communication of caring and (3) reassurance of HIV care and prevention services’ trustworthiness. We take these functions to be integral to the combination's mechanism of effect.

The few existing research reports examining follow-up through home visits as a linkage intervention offer glimpses into intervention dynamics, briefly referencing the educational function of counselling as part of follow-up [22], and/or its encouraging role in overcoming barriers [26,27]. One qualitative study highlighted inadequate counselling on “when and why” infected persons should make the link to care (our “orientation and guidance”) as a linkage barrier. This led to the development of an extended counselling intervention based on monthly home follow-up visits. Intervention recipients were almost twice as likely as controls to link to care after three months [26], and 2.5 times more likely to be retained in pre-ARV care at 24 months [28]. A recent study examining follow-up by peers and lay persons in retaining HIV-infected Ugandan women in post-natal care points to “feeling comfortable” disclosing HIV-positive status to other infected persons as important for intervention effectiveness [29].

A number of questions emerge from our analysis, which might be addressed in future research. In highlighting functions, we have traced specific and differing contributions to linkage, while also showing that the contributions of improved morale, communication of caring, and reassurance of services’ trustworthiness, cut across intervention components. Recognizing the common functions of intervention components suggests a reinforcing and integrating effect of using them in combination, and indicates the “whole” of the combination may be more than the simple sum of its parts. How can the synergies inherent in combination interventions be brought to light and better understood?

The question of how The Linkages Study intervention functions might be integrated into public health services also deserves consideration. Like much of Africa, Uganda has a decentralized public health system that includes community-based services. Village Health Teams (VHTs) are made up of community health workers, lay members of communities who volunteer to provide services intended to “bridge the gap” between communities and the formal health system [30]. Working at the village level with support from implementing partners, VHTs promote use of health services and make referrals, including referrals for HIV testing. With additional training and support, might Ugandan VHTs also carry out community-based HIV testing and in-home follow-up?

This qualitative study has the following limitations. We have worked to represent study results in broad language that will be meaningful in other settings; however, as qualitative research, results are not strictly generalizable. Also, no direct data on counselling interactions, such as observations, were collected, precluding the triangulation of data sources and claims about what “actually” took place in counselling interactions. Finally, we were not able to examine every intervention component; the contributions of text message reminders are not represented here.

## Conclusions

To realize the potential of community-based testing initiatives in Africa, following testing individuals should be able to link promptly to effective HIV care, treatment and prevention services. Until services are routinely available in communities, access means attending clinics. To accomplish this, many would benefit from additional support.

**Figure 1:**
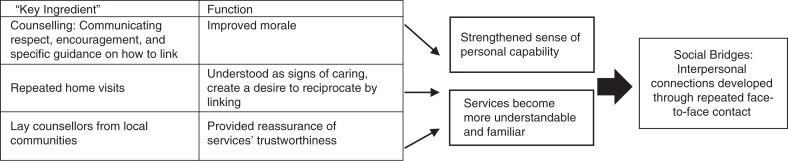
How lay counsellor home HTC with follow-up support leads to linkage to clinic-based HIV treatment and prevention services.

Lay counsellor home HTC with follow-up provides this support by building “social bridges” – interpersonal connections established and developed through repeated face-to-face contact between lay counsellors as service representatives, and prospective service users (Figure 1). Social bridges link communities to the service system, inspiring individuals to overcome obstacles and access care.
